# The Gift of Expression

**Published:** 1920-10-16

**Authors:** 


### THE EDITOR'S LETTER-BOX.

**Correspondence on all subjects is invited, but we cannot in any way be responsible for the opinions expressed by our correspondents, who must give their name and address as a guarantee of good faith, but not necessarily for publication. Correspondents are reminded that brevity of style and conciseness of statement greatly facilitate early insertion.**

### "The Gift of Expression."

*To the Editor of* The Hospital.

Sir,—As a member of the rank and file I should like to convey to the writer of the article, "The Gift of Expression," our appreciation and thanks for that, and her incisive reply to the indignant protest it called forth from a College member. We also owe a debt of gratitude to this other emphatic lady for having, by her choice of analogy, played the game into our hands. She rather refutes her own argument in taking for example the case of teacher and taught. If a class is apathetic and indifferent the reason is obviously one of two: either those responsible for the physical well-being of the class are at fault or there is failure of some sort on the part of the teacher. We don't expect to hear much about it from the teacher certainly; but what about those responsible for the teacher? Would they be fair to the pupils in keeping silence, and have not the pupils themselves something of a case to defend?

There are many of us, I think, who would, if we could, express the same thoughts in the same terms as the writer of the article, and we are quite aware that the accusation it contains is only too well-founded.

And is the training-school to blame?

Undoubtedly to a great extent it is. It is common knowledge that the nurse at the end of her training is a very weary woman indeed. In her three or four years of immersion in hospital she has lost touch, except in a very superficial degree, with contemporary social questions and, unfortunately, in some measure with her own people and friends. Her interests have come to be in common with those of her fellow-worker, and are bounded by the four walls of the hospital. Life has been school again in earnest—a hard drilling in routine, discipline (according to local traditions good, bad, or indifferent), endurance, and self-repression. The work, of course, is absorbing, and the daily round—or, rather, the daily or nightly race with time—has its own peculiar fascination. But the nurse's individual tastes and aspirations have no encouragement there, and mental and physical tiredness, coupled with lack of time and opportunity, make any effort to keep abreast of the times all but impossible. In short, her perspective has had to adjust itself to correspond with the limitations of her very narrow existence. She emerges from her training-school totally unaware of, and but half-equipped for, the struggle that awaits her, and some measure of that struggle may be gauged by studying the advertisement columns of the nursing and other papers.

Possibly there is much that one could not and would not alter in the old system, but there is also much that will have to be scrapped because times have progressed fast during and since the fateful period of war, and we have to adapt ourselves to new needs and new social conditions. There are certainly signs of better things in store for us all, but particularly perhaps the rising generation: schemes for organisation and education, reforms dealing with hours on duty and conditions of working. The system of the "living-out" nurse or probationer has—thanks to the far-seeing guardians of the Lambeth Infirmary—introduced the thin end of the wedge, and all these changes will combine in time to clear up many difficulties.

In the meanwhile, however, there is but one hope of remedy for the failure of present and passing generations of the rank-and-file to grasp, and make good, their opportunities—namely, what we may call the "house-tops" method. By all means—even at the risk of its having a somewhat depressing effect on the angels—let us hear of our manifold failings from the house-top or any other eminence. What are our leaders for? Is it not encouraging that it has stimulated one member already into response! Besides, the press flatters itself that it is *vox populi*, a fact we might have realised and made use of, to our advantage, long ago.

It would seem that our friend, the writer of the article, has the voice and the power to use it for our good and enlightenment, and we hope to hear it again and again till we are roused from our apathy and quickened to a livelier sense of our obligation, to ourselves, to one another, and to the future of the whole profession, no less than to the sick, for whose welfare we are continually and—in justice to ourselves be it said—unobtrusively striving.

A Middle-aged Member of the Rank-and-File.

### The Great Northern Appeal.

*To the Editor of* The Hospital.

